# The Microbe of Yellow Fever

**Published:** 1887-06

**Authors:** J. McF. Gaston


### THE MICROBE OF YELLOW FEVER.

The following article and note accompanying it were received by us too late for insertion in the appropriate department of this issue of the RECORD:

### ON THE MICROBE OF YELLOW FEVER AND ITS ATTENUATION. BY DR. DOMINGOS FREIRE, DR. PAUL GIBIER, AND DR. C. REBOURGEON.

In a previous communication, presented to the Academy of Sciences in November, 1887, we announced the discovery by one of us of a microbe found in the blood, both in the ejecta and the organs, of individuals who had died of yellow fever. We described its form and mode of evolution in broths wherein it had been cultivated, and, finally, the attenuation of its virulence in these same broths, and its inoculation as a prophylactic means. Since that time we have continued those researches which better perfected means of investigation have enabled us to bring about.

When one examines through the microscope the blood of an individual who has been driven to the last stage of yellow fever, one perceives a pretty good number of very fine, brilliant and movable micrococci circulating between blood corpuscles. These same micrococci are also found, and in still greater quantity, in the stomachal mucous, in the substance of negro vomitu, in the urine, and in the yellowish and viscid matter of the intestines.

If, by means of a sterilized pipette, we draw from the heart of an individual who has just died of yellow fever a small quantity of blood and drop it in the culture fluid, we observe the following: On the first day the broth becomes ill-colored, the blood corpuscles settle at the bottom of the bowl. On the following days the broth becomes more and more opaque, first whitish, then yellowish at the surface, depositing at the fundus and on the sides to which it adheres a substance at first of a caseous appearance, but soon assuming a shade varying from light-brown to deep-black ; moreover, at this point, there arises from the bowl an odor *sui generis*, bringing forcibly to mind that of black vomit.

The microscopical examination of this broth elicits a considerable proliferation of micrococci identical with those that are found in morbid liquids. They can be seen clinging to each other so as to form long, tiny chains, flexuous, movable and distorted in every direction.

If, by means of a platinum wire, one plants, as it were, this broth in a solid medium susceptible of cultivation, the development of the micrococcus is effected in a colony assuming the form of a nail whose point is sunk in the gelatinous mass and whose head spreads on the surface. This culture-fluid presents a singular appearance. The product is white, brilliant, glossy ; it liquefies the gelatin slowly. Various shades of aniline, particularly the chlorydrate of rosaniline and the violet of methyl, easily color the micrococcus.

The chemical examination of the blackish material constituting the deposit found at the bottom of each bowl indicates that they contain ptomaines similar to those of black vomit.

We will give in a special communication a study on the ptomaines of yellow fever and their toxicology.

The transmissibility of yellow fever to animals is possible through the injection of morbid material and through culture fluids, particularly to rabbits, cobayes and birds. A long series of experiments enable us to state that fact.

Other experiments following the same steps have had identical results. Dr. Range, physician (*premiere classe*) in the Marine, in a long report on an epidemic of yellow fever in the Islands of Guiana, claims to have transmitted the virus to animals by inoculation. He adds, that means of investigation failed him to define the form of the microbe which he had discovered in the blood of the infected. Quite recently, Dr. Finlay, in a communication published in the *Scientific Review*, has given the description of a micrococcus which he found in the blood of individuals sick with yellow fever. He also cultivated it in solid media, and the description which he gives thereof corresponds to the characters of our preparations. Dr. Maurel, principal physician of the Marine, has also seen micrococci in the blood of persons afflicted with yellow fever.

Observation has demonstrated to us that the virulence of culture-broths did not last beyond eight or ten days. From that moment, if one continue to inject a certain quantity of this broth in rabbits or cobayes, these animals cease to succumb ; on the contrary, they secure immunity. It has, therefore, been an easy task to prepare broths of different degrees of attenuation, in order to convert them into harmless vaccinating matter. We will add that the first broth is always more virulent than blood itself, but its virulence slackens from the seventh or eighth day. On this point our experiments are very numerous. We have already described them in our first communication to the Academy of Science in 1884. Before concluding, we must remind our readers that, as a sequence of the operation of Dr. Domingos Freire on the attenuation of the *amaril virus*, inoculations with attenuated cultures have been made in Rio de Janeiro on *several thousand* individuals.

We will present in a very short time, to the Academy, statistics backed by official documents and showing results obtained through these vaccinations.

MESSRS. EDITORS : The foregoing paper has been kindly translated from the French by Dr. E. van Goidtsnoven at my request, being presented by the authors to the Academy of Science in the Institute of France, at Paris, and sent to me by Dr. Domingos Freire on April 12, 1887.

As this embodies a summary of the most advanced developments connected with the preventive inoculation against yellow fever, it seems opportune that the medical public should be in possession of these points when our Government has authorized an investigation of the claims of yellow fever inoculation in Mexico and Brazil.

Believing that all which has been claimed for this prophylactic measure will be verified, I await the scrutiny with confidence in the result.

J. McF. GASTON, M D.

